# The association between extremely low-frequency electromagnetic fields and childhood leukaemia in epidemiology: enough is enough?

**DOI:** 10.1038/sj.bjc.6605837

**Published:** 2010-09-28

**Authors:** S Schmiedel, M Blettner

**Affiliations:** 1 Institute for Medical Biostatistics, Epidemiology and Informatics (IMBEI), University Medical Center of the Johannes Gutenberg University Mainz, Mainz 55101, Germany; 2 Danish Cancer Society, Institute for Cancer Epidemiology, Copenhagen 2100, Denmark

Two articles investigating the association between exposure to extremely low-frequency electromagnetic fields (ELF-EMF) and the risk of childhood cancer, especially leukaemia, are published in this issue of the *British Journal of Cancer*. The first article (Kroll *et al*, 2010) was based on data from an older study published by Draper *et al* in 2005 and used data from 1962 to 1995. In the publication of Draper *et al*, distance from the next power line to the place of residence of cases and controls was the exposure measure, whereas Kroll *et al* used a refined exposure measure based on field calculations. These were performed for houses at distances closer than 600 m (458 of 58 162 addresses) to power lines. However, for 25% (116) of the addresses that were close to a power line, field calculation was not possible. Results of different analyses of the same data set (Draper *et al*, 2005; Kroll *et al*, 2010) are rather similar. Draper *et al* found a significantly elevated risk (⩽200 *vs* >600 m, odds ratio (OR): 1.69, 95% confidence interval (CI): 1.13–2.53) in persons living close to power lines. With the new exposure measure, Kroll *et al* also found an increased risk for leukaemia in the highest exposure group (⩾0.4 *vs* <0.1 *μ*T, OR: 2.0), although not statistically significant (95% CI: 0.18–22.04).

The data from the study by Kroll *et al* were also part of a pooled reanalysis, which is the second article on the subject in this issue. This was based on a pooled reanalysis of seven studies published after 2000 on leukaemia in children aged 0–15 years (Kheifets *et al*, 2010). The study included 10 865 cases and 12 853 controls and the data of Kroll *et al* contributed to the majority (89%) of cases. Although not statistically significant, an increase in risk of leukaemia by increasing exposure to ELF-EMF was found (⩾0.3 *vs* <0.1 *μ*T, OR: 1.44, 95% CI: 0.88–2.36). In the pooled analysis, exposure categorisation was different, and it is noticeable that results of the analysis by Kroll *et al* seem completely different in the pooled reanalysis by Kheifets *et al.*

First, there are 12 more cases and 24 more controls in the pooled analysis than in the original publication. This is because matching was ignored in the pooled reanalysis and therefore more cases and controls could be included. Second, different ranges of exposure classification were used, which yielded a different category for *one* control, which had a large impact on the results of the study. Kroll *et al* found an increased OR of 2.0 (two cases *vs* one control) for the category above 0.4 *μ*T, whereas Kheifets *et al* reported an adjusted OR of 0.98 for the category above 0.3 *μ*T (one case *vs* one control). This shows how unstable results are if numbers are small, and the danger of using different cutoff points in different analyses.

Although both studies are state of the art, one may question why a pooled analysis was performed if about 90% of the data originated from a single study. Results do not contradict epidemiological knowledge from older investigations, which were summarised in two reviews by Ahlbom *et al* (2000) and Greenland *et al* (2000). However, the estimated risk in the current pooled analysis is slightly lower than that reported by Ahlbom *et al* (2000) (OR: 2.00, ⩾0.4 *vs* <0.1 *μ*T) and by Greenland *et al* (2000) (OR: 1.7, ⩾0.3 *vs* <0.1 *μ*T).

Over three decades, epidemiological studies have investigated the association between leukaemia and ELF-EMF. The first studies had several methodological limitations, and results might be biased. More recent studies have certain methodological advantages, especially an improved ELF-EMF exposure measure. However, they resulted in comparable risk estimates, as shown in the pooled analysis.

In summary, epidemiological studies have repeatedly shown positive results for this association. However, a causal biological mechanism for carcinogenicity of EMF-ELF has not yet been found, despite intense research (WHO, 2007, Chapter 11.3.6). Thus, the epidemiological results stand alone. The International Agency for Research on Cancer (IARC) has categorised ELF magnetic fields in 2002 as being possibly carcinogenic (IARC, 2002) only on the basis of concordant findings in epidemiologic studies. The categorisation was not changed by the World Health Organization (WHO, 2007, Chapter 11.5), which included more recent studies.

We believe epidemiology cannot provide any further evidence. On the basis of current knowledge on possible causal mechanisms and epidemiological methods, better insights into this association cannot be expected. Hence, a point in the discussion by Greenland *et al* (2000) that ‘possibly […] measures only reflect effects of some biologically relevant exposure that is missing from […] data.’ is still valid after 10 years, despite many additional published studies.

We agree with the ideas mentioned by Savitz (2010) in his article entitled ‘Aetiology of epidemiologic perseveration: when enough is enough.’ As long as no emerging new ideas become apparent (e.g., better exposure assessment, biological mechanism, important confounders), we should accept the limits of epidemiological research. This is mainly true, as the percentage of highly exposed children is below 1%, and the public health impact is low.
